# Nano-antenna enhanced two-focus fluorescence correlation spectroscopy

**DOI:** 10.1038/s41598-017-06325-6

**Published:** 2017-07-20

**Authors:** Lutz Langguth, Agata Szuba, Sander A. Mann, Erik C. Garnett, Gijsje H. Koenderink, A. Femius Koenderink

**Affiliations:** 1 Center for Nanophotonics, AMOLF, Science Park 102, Amsterdam, NL-1098XG The Netherlands; 2 Biological Soft Matter Group, AMOLF, Science Park 102, Amsterdam, NL-1098XG The Netherlands

## Abstract

We propose two-focus fluorescence correlation spectroscopy (2fFCS) on basis of plasmonic nanoantennas that provide distinct hot spots that are individually addressable through polarization, yet lie within a single diffraction limited microscope focus. The importance of two-focus FCS is that a calibrated distance between foci provides an intrinsic calibration to derive diffusion constants from measured correlation times. Through electromagnetic modelling we analyze a geometry of perpendicular nanorods, and their inverse, *i.e*., nanoslits. While we find that nanorods are not suited for nano-antenna enhanced 2fFCS due to substantial background signal, a nanoslit geometry is expected to provide a di tinct cross-correlation between orthogonally polarized detection channels. Furthermore, by utilizing a periodic array of nanoslits instead of a single pair, the amplitude of the cross-correlation can be enhanced. To demonstrate this technique, we present a proof of principle experiment on the basis of a periodic array of nanoslits, applied to lipid diffusion in a supported lipid bilayer.

## Introduction

Fluorescence correlation spectroscopy is a common technique to deduce the concentration and mobility of fluorescent particles. It is based on measurements of fluorescence intensity fluctuations, which occur as particles perform a random walk through a single, tight, microscope focus^1–3^. These intensity fluctuations are correlated on a time scale comparable to the time required to diffuse through the focus, and are especially prominent for concentrations lower than one fluorescent particle per focus volume. Apertures in metallic films^4, 5^, bull’s eye antennas^6, 7^, and nanoparticles with plasmonic resonances^8–11^, have been demonstrated as a means to reduce focus size, thereby significantly extending the concentration range of FCS, even to biophysically relevant micromolar concentrations^12^. In addition to possible orders of magnitude reduction in detection volume, plasmonic nanostructures can also dramatically improve fluorescence count rates by enhancing radiative emission and redirecting light^7, 13^, Since count rates enter quadratically as a reduction in FCS acquisition time, signal enhancements are highly useful.

A main drawback of FCS is that conversion of a measured correlation time into a diffusion constant requires accurate knowledge of the focus size and shape^14, 15^, Aberrations or imperfect alignment of confocal pinholes can significantly change the measured properties^14^. In standard FCS protocols it is therefore necessary to perform calibration measurements on samples of known kinetic properties^16^. In the case of nano-antenna enhanced FCS the need for calibration is even stronger, as the detection volume depends on the optical properties of the antenna at the pump and fluorescence wavelengths, and even the fluorophore quantum efficiency^13^ and rotational diffusion time of the diffusing species. This makes it challenging to perform proper calibration, because a reference specimen is required with exactly the same photophysical and similar hydrodynamic properties.

In this paper we propose dual focus nano-antenna FCS. In conventional dual focus FCS (2fFCS), the intensity fluctuations originating from two spatially well separated diffraction limited foci are cross-correlated^17, 18^. Two-focus FCS is a robust method to measure absolute diffusion coefficients, as the distance between the two detection volumes can be precisely set in an experiment. This distance serves as length calibration, independent of aberrations^17, 18^, Here we explore if we can mitigate or eliminate the problematic calibration of nano-antenna enhanced FCS by utilizing multiple foci, as in 2fFCS, while maintaining the benefits provided by nano-antennas^19^. This paper is structured as follows: first, we design geometries for 2fFCS based on polarization multiplexing. We then show that while nano-particle antennas are not suited, nano-apertures of alternating orientation should give a distinct two-focus signal. Finally we present an experimental proof of principle in the context of lipid diffusivity in model biomembranes.

## 2fFCS requirements

In FCS, one measures the normalized time-correlation of (fluctuating) detected intensities as given by:

1 $${G}_{i,j}(\tau )=\frac{\langle {I}_{i}(t){I}_{j}(t+\tau \rangle )}{\langle {I}_{i}(t)\rangle \langle {I}_{j}(t)\rangle },$$

where 〈·〉 indicates averaging over time *t*, while *τ* indicates the particular time-delay value at which one evaluates the temporal correlation. We have introduced subscripts *i* and *j* as labels for detected intensities on possibly distinct detectors. In standard FCS one uses a single detection channel (*i* = *j* = 1), whereas in 2fFCS one can measure the autocorrelation (*i* = *j*) or cross-correlations *i* ≠ *j* between two detectors^2, 14, 17^. It can be shown that the correlations should be equal to:

2 $${G}_{i,j}(\tau )-1=\frac{\int d{\bf{r}}\int d{\bf{r}}^{\prime} MD{F}_{i}({\bf{r}}){G}_{D}({\bf{r}},{\bf{r}}^{\prime} ,\tau )MD{F}_{j}({\bf{r}}^{\prime} )}{{C}_{0}{(\int d{\bf{r}}MD{F}_{i}({\bf{r}})\int d{\bf{r}}MD{F}_{j}({\bf{r}}))}^{2}}$$

where *G*_*D*_(**r**, **r′**,*τ*) is the diffusion kernel that quantifies the probability for a molecule to diffuse from **r′** to **r** in a time *τ*, and *C*_0_ is the concentration. *MDF*_*i*_ is the molecular detection function, which indicates the probability that a molecule at **r** actually gives rise to a photon detection event in detection channel *i*. In 2fFCS one uses two spatially separated molecular detection functions *MDF*_1_(**r**) and *MDF*_2_(**r**), which originate from two displaced foci. Fluorescence events from these foci are detected either via two separate detectors, confocal with each excitation focus, or via a temporal multiplexing scheme in the excitation beam^14, 17, 18, 20, 21^.

In 2fFCS, one expects the temporal cross-correlation (*i* ≠ *j*) between two detection channels to differ significantly from the autocorrelations (*i* = *j*). In particular, for well separated detection volumes, the autocorrelation shows a distinct peak at a characteristic time *τ*_*C*_ that directly derives from the focus separation. If the center-to-center distance *R* between the foci is known, the diffusion constant can be determined directly from *τ*_*C*_. However, it should be noted that a clear peak in the cross-correlation function is not strictly necessary for 2fFCS to be useful: if the shape of the *MDF* s is known, simultaneous fitting of auto- and cross-correlations also allows accurate determination of the diffusion constant. For example, Dertinger *et al*.^17, 18^, derived explicit FCS trace dependencies assuming Gaussian foci, and argued that the height of the cross-correlation contribution decreases with *R* ^−3^ or *R* ^−2^ in the case of diffusion of analytes in 3D or 2D, respectively.

**Figure 1:**
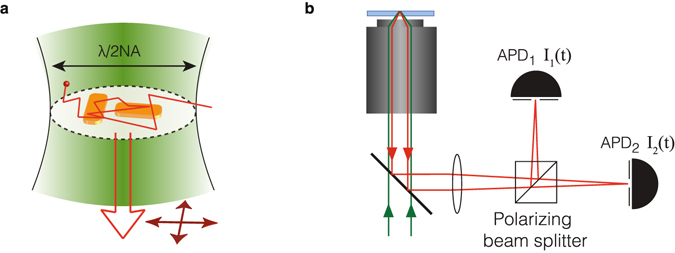
We propose a nano-antenna version of dual focus FCS based on polarization encoding the fluorophore position near plasmonic nano-antennas. (**a**) Two plasmon antennas with an orthogonally polarized resonance are placed in the diffraction-limited focus of a fluorescence confocal microscope. When diffusing fluorophores are near an antenna, the otherwise unpolarized fluorescence becomes polarized along the antenna resonance. (**b**) Fluctuating intensities on two APDs that are confocal with both antennas, but sensitive to orthogonal polarizations, will show a temporal cross-correlation. The antenna spacing acts as a ruler for measuring diffusion constants.

Nano-antenna enhanced 2fFCS can in principle give closely spaced detection volumes with small overlap. However, if one would place a plasmonic 2fFCS substrate in the focal plane of a conventional microscope, the far field optics typically can not resolve the two foci at subwavelength distance. We propose that nano-structures can encode the spatial origin of fluorescence emission into two orthogonally polarized detection channels, as sketched in Fig. 1. Key to this encoding is that one designs a structure composed of antennas with a strongly linearly polarized response of orthogonal orientation for distinct emitter positions. The antennas should then be aligned with two far-field detection polarization channels. In this work we discuss two of the simplest nano antenna geometries that provide a strong polarization response: nanorods, and their inverse, *i*.*e*., nanoslits in a metal film. We focus on designs to measure diffusion in 2D systems, such as lipid bilayers^12, 22^, that can be draped over a plasmonic surface.

## Numerical approach

Before we present numerical results on nano-antenna enhanced 2fFCS, we outline the general calculation approach. Consistent with reported plasmon FCS results^4–7, 9–13^ we choose gold as a plasmonic material, and therefore design the antennas to work in the long-wavelength part of the visible spectrum, around 650 nm. In a typical setting these antennas would be fabricated on glass, and embedded in water that accommodates the lipid bilayer. For sake of concreteness we present results for a 2D diffusion coefficient of *D* = 4.5 · 10^−8^ cm^2^/s at a surface concentration of *C*_0_ = 1 · 10^−13^ m^−2^, appropriate for diffusion in supported lipid bilayers. We assume the antennas to be covered by a thin dielectric planarizing layer, providing a flat plane for the diffusing lipids 30 nm above the metal interface.

We designed antennas to obtain a resonance that is broad enough to cover both excitation and emission wavelengths, while giving a low response in the orthogonal polarization. To numerically investigate the performance of these antennas for 2fFCS, we first need to estimate the molecular detection function. The *MDF* is in essence given by the excitation efficiency function (EEF) and the collection efficiency function^2^ (CEF): *MDF*(*x*, *y*) = *EEF*(*x*, *y*, *z*_0_) · *CEF*(*x*, *y*, *z*_0_). The EEF is the probability to excite a fluorophore at a given position, and scales linearly with the pump field intensity. In an experiment unpolarized or circularly polarized light should be used to ensure that excitation close to both nano-rods or slits occurs with equal probability. Hence, for the EEF we take the sum of the excitation field intensities for both linear polarizations at the pump wavelength (specified below for the two case studies) as calculated with full wave simulations. This approach ensures that the excitation field is taken into account, which is crucial, as the exciting beam can lead to background intensity strongly affecting results^23^.

The near-field resulting from an incident beam at the emission wavelength instead of the excitation wavelength can provide us with the collection efficiency *CEF* ^24^. Through reciprocity, the calculated near-field at a location near the antenna provides the power one would collect in the far field from a classical constant-current source at that location^25^. Using the near-field intensities upon polarized excitation as collection efficiency functions (*CEF* s) we obtain:

$$\begin{array}{rcl}MD{F}_{X}(x,y,{z}_{0}) & = & EEF(x,y)\cdot {I}_{E,X}(x,y,{z}_{0})\\ MD{F}_{Y}(x,y,{z}_{0}) & = & EEF(x,y)\cdot {I}_{E,Y}(x,y,{z}_{0}){\boldsymbol{,}}\end{array}$$

where *I*_*E*_ is the electric field intensity in the *x*, *y* plane at *z*_0_. It should be noted that this approach assumes randomly oriented fluorophores, implying rapid rotational diffusion compared to the fluorescence decay time, which is valid for typical fluorophores (rotation diffusion times about one order of magnitude shorter than decay times)^3, 26^. Finally, the molecular detection functions can be converted into simulated FCS time traces by numerically executing the integration in Eq. (2), with the assumption of a membrane-diffusion application which limits integration to a 2D plane.

## Numerical results for nanorods

### Nanorods

**Figure 2:**
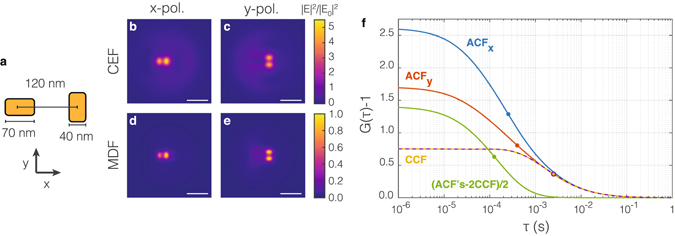
Nanorods for nano-antenna dual focus fluorescence correlation spectroscopy. (**a**) A sketch of the nanorod based geometry for nano-antenna 2fFCS. Two 40 × 70 nm nanorods (30 nm height) with 120 nm center to center distance form a T-shape. When placed on glass and in water, their near field response is resonant at 676 nm. (**b**,**c**) The near-field intensity in a plane 30 nm above the rods, upon plane wave illumination at 690 nm (targeted emission wavelength) for polarization along the horizontal (**b**) and vertical (**c**) rod. Through reciprocity, this intensity is proportional to the collection efficiency function. The scale bar is 200 nm. (**d**,**e**) The molecular detection efficiency reconstructed from simulations for a *λ* = 676 nm pump wavelength (*w*_0_ ≈ 300 nm Gaussian beam width) and *λ* = 690 nm emission wavelength (i.e., panels b,c). As a global scaling factor does not affect the correlation curves, both MDFs are normalized to the maximum in panel d). (**f**) Predicted autocorrelation functions for the intensity on the *x*- and *y*-polarized detector (blue and red curves), predicted cross-correlation between polarization channels (yellow and dashed purple), and autocorrelation of the polarization contrast (green), constructed as (*ACF*_*x*_ + *ACF*_*y*_ − 2*CCF*)/2. Circles indicate the time at which the correlation has dropped to 50% of its maximum value at *τ* = 0.

As the simplest polarization sensitive geometry, we use a nanorod geometry. Gold (tabulated optical constants from Johnson and Christy^27^) nanorods in water (*n* = 1.33) on glass (*n* = 1.45) can be matched to the resonances of red-fluorescent dyes such as the commonly used Alexa647. In the numerical examples here we will use an excitation wavelength of 676 nm and an emission wavelength of 690 nm. Accordingly we focus on gold nano-rods of 70 nm length, 40 nm width, and 30 nm height which have a resonance at ≈676 nm vacuum wavelength but is broad enough to cover both the excitation and emission wavelength. If we place a second nanorod at right angles to the first (see Fig. 2a), one obtains a configuration that upon different incident far-field polarizations gives rise to near-fields at distinctly different spatial locations, localized at the tips of the accordingly oriented nano-antenna. Conversely, emission from sources located in the hot spots at either rod is expected to be strongly polarized along the adjacent rod due to coupling of the transition dipole with the particle resonance^24, 28^. It is important to position the rods in such a way that cross-talk between polarization channels is minimized, as polarization cross-talk will degrade the 2fFCS crosscorrelation contrast. For minimum polarization-crosstalk it is advantageous to aim for minimum near-field coupling between the plasmon particles. At least to first order, in a simple picture of coupled dipolar resonances, this is achieved by placing one rod exactly along the symmetry axis of the other. As actual FCS experiments are sensitive to near-field detail, full wave simulations are required to calculate the actual fields and MDFs, thereby fully accounting for any coupling that may occur between the antenna. We perform full wave simulations using the finite-difference time-domain method (Lumerical FDTD Solutions^29^), and report the resulting near-field intensity maps in Fig. 2b and c. Here we used a gaussian beam with an NA = 0.7 (beam waist *w*_0_ ≈ 300 nm) to excite the rods with polarizations aligned along both of the rods. The rods are separated by 120 nm center-to-center (see Fig. 2a), and light is incident from the glass side. It should be noted that in addition to the localized fields there is substantial background electromagnetic energy density, due to the beam focus. We construct the CEF and MDF from these gaussian beam simulations (at 676 and 690 nm wavelength) as input in Eq. ((2)). The obtained molecular detection functions (Fig. 2d,e) are strongly peaked at the rod ends, and clearly spatially distinct.

Figure 2f reports the resulting simulated fluorescence correlation traces. First, one notes that intensity autocorrelation functions (*ACF*) of the two polarization channels give the typical roll-off behavior expected for FCS. As the *MDF* s for *x* and *y* polarization are not identical due to the structural asymmetry, the *ACF* s also show different zero-time contrasts (which is a measure for the MDF-volume). Their roll-off times are 2.5 ms in case of *x*-polarized light and 3.5 ms for the *y* polarization, as indicated by the dots in Fig. 2f. The corresponding length scale $(\sqrt{4D\tau })$ of around 210 to 250 nm is close to the diffraction limit rather than being close to the antenna hot spot size, commensurate with the finding that nano-antenna FCS can only outperform conventional diffraction-limited FCS if the hot spots are sufficiently strong compared to the background focus field^23^.

Figure 1(d,e) shows that cross-correlating the two detectors is actually predicted to give an FCS trace similar in shape to the autocorrelation functions, though lower in contrast. Importantly, there is no evidence of a distinct peak in the autocorrelation at a characteristic non-zero *τ*_*C*_, and the cross-correlation is monotonically decreasing. This result is indicative of insufficient spatial separation between the *MDF* s, as a consequence of the large contribution of the (overlapping) background intensity. This is a known problem that also negatively impacts simple nano-antenna enhanced FCS with nanoparticles^23^. The reader should be warned that one easily underestimates the role of the background focus in spatial maps as shown in Fig. 2(b–e). However, it is not the contrast in *peak field intensity* in Fig. 2 that matters, but rather the area-integrated content. Even at the large calculated contrast, the large diameter of the focus compared to the hot spots means that the background light will contribute significantly. This observation implies that even though the spatial maps of the polarization-resolved MDFs locally show large contrast at the antennas, the non-zero polarization-agnostic background implies strong cross-talk between detector channels. If one would not correlate intensity traces, but rather measure the temporal correlation function of instantaneous polarization differences by autocorrelating *I*_*x*_ − *I*_*y*_ (in practice obtainable through *ACF*_*x*_ + *ACF*_*y*_ − 2*CCF*), one finds the shortest roll-off time of 1.1 ms (red dot in Fig. 1). This corresponds to the time over which the emission polarization of randomly oriented fluorophores coupled to one of the antennas is conserved. It is therefore a measure for the diffusion time through the near-field of a single antenna. Hence, a plasmon nano-rod antenna geometry does provide two well-separated hot spots that are addressable by orthogonal polarizations. However, due to the background focus that enters both the *MDF* s, cross talk dominates the detector cross-correlation.

### Nanoslits

**Figure 3:**
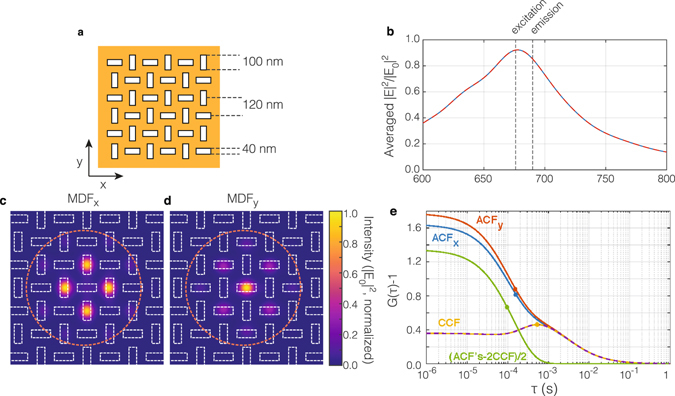
Periodic arrays of plasmonic nano-apertures for fluorescence correlation spectroscopy. (**a**) Sketch of an array of nano-apertures on a square grid with pitch *d* = 120 nm. The apertures alternate in orientation and measure 100 by 40 nm. The apertures are assumed located in a 100 nm thick gold film on glass, immersed in water. (**b**) For both *x*- and *y*-polarized incident light, the near-field in a plane 30 nm above the film (averaged over unit cell) shows a peak at 670 nm. (**c**,**d**) The calculated MDF’s for unpolarized excitation at 670 nm, and *x*– and *y*– polarized detection, assuming Gaussian beam optics with an NA of 0.7. The focused beam waist is shown with a dashed orange circle, and the 100 × 40 nm apertures are outlined on top of the color maps in white. Importantly, the MDFs are localized on subsets of differently oriented holes. (**e**) The autocorrelation functions for the intensity measured by the *x*- and *y*-polarized detectors (blue and red curves), the predicted cross-correlation between polarization channels (yellow and dashed purple curves), and autocorrelation of the polarization contrast (green, calculated as (*ACF*_*x*_ + *ACF*_*y*_ − 2*CCF*)/2). The spatial overlap of the two *MDF* s is sufficiently small that the cross-correlation shows a distinct maximum at 0.53 ms. The dots depict the roll-off time where the correlation has dropped to 50% of its maximum value (ACF), resp. the CCF peak position.

Having identified that 2fFCS requires not just localized hot spots, but also efficient suppression of background intensity, we propose two modifications. First, a nano-slit or nano-aperture geometry that uses apertures in thick metal films effectively blocks background signal, as already shown for single-focus antenna-enhanced FCS^4, 7, 30^. Second, by making the structure periodic, one can increase the amplitude of the cross-correlation. The proposed structure (shown schematically in Fig. 3a) consists of an optically thick gold-film (100 nm) perforated by rectangular nano-apertures. We study an arrangement on a square grid of deeply sub-diffraction pitch, with the orientation of apertures alternating along the *x* and *y* axis, such that neighbors always have opposite orientations. This periodic arrangement has the advantage that detecting an emitter in an aperture of opposite orientation does not require diffusion from one aperture to another but from one aperture to any of four others, which are all located at the same distance. This increases the cross-correlation term by a factor of four, but keeps its shape and the peak position the same. It should be noted that diffusion beyond-nearest neighbor holes adds a long-time tail, also accounted for in our work, without changing the shape of the short-time cross correlation contribution.

For the periodic array of nano-apertures we again perform full wave simulations with FDTD using a Gaussian excitation beam corresponding to a tight focus (NA = 0.7), and we obtain fields at the optical pump wavelength and Stokes-shifted fluorescent wavelength (taken as 676 nm resp. 690 nm). Figure 3b shows the averaged near field intensity spectrum 30 nm above the gold film, with the excitation and emission wavelengths indicated with dashed lines. A clear resonance is visible at the excitation wavelength. Figure 3c,d report the *MDF* for two orthogonally polarized detectors, assuming that the FCS sampling plane is again 30 nm above the gold surface. Fields plots (not shown) show that the nano-aperture respond strongly when oriented perpendicular to the driving field polarization, as expected according to the Babinet-principle^31–33^. Commensurate with the result that the nanorods have a strongly polarized resonance, the *MDF* is high at nano-apertures oriented perpendicular to the detection polarization, yet low at the other apertures. The cross talk between *MDF* s is small, owing to the fact that the metal film blocks light, and resonances have field intensity peaks localized right above the aperture.

The correlation functions calculated for the nano-aperture array are shown in Fig. 3e. The autocorrelations for each polarization channel are slightly different because in the simulation the focus is centered on a vertical slit. The autocorrelations vary slightly with excitation beam position. Due to the presence of multiple displaced detection volumes, the *ACFs* do not roll-off monotonically, but show shoulders near 1 ms. The roll-off time (point where the ACF *G*(*τ*) − 1 has reduced to half its maximum) is 0.16 ms for both polarizations (blue and red dots). The cross-correlation functions (yellow and purple) show a distinct peak at *τ*_*peak*_ = 0.533 ms, proving that the nano-aperture design indeed provides a sufficiently small spatial overlap between the *x*- and *y*-polarized *MDF* s to make dual focus cross-correlation FCS possible. The autocorrelation of the polarization contrast Δ*I* (green curve) yields a roll-off time of 0.096 ms, corresponding to the diffusion time through a single aperture. Despite the fact that now multiple hot-spots are present, the individual detection volumes are smaller than in the nanorod geometry thanks to the reduced background intensity. As a result, the correlation contrast is higher.

If the 2fFCS cross-correlation shows a peak at non-zero delay time, the peak-time can be converted in a diffusion constant. For 2fFCS experiments performed with two displaced Gaussian foci (identical size *σ* and separation *R*), one would expect a peak correlation at a delay *τ*_*peak*_ = (2*R* ^2^ − 2*σ* ^2^)/(8*D*)^17, 18^. If we take the roll-off time of the polarization fluctuations (sum of autocorrelates minus cross-correlates; Fig. 3e, green line) of *τ*_Δ*I*_ = 0.096 ms as a measure for the size *σ* of the individual hot-spot through *σ* ^2^ = 4*τ*_Δ*I*_ *D*, and knowing the set distance between the two *MDF* s (*R* = 120 nm), one retrieves the diffusion coefficient as *D* = *R* ^2^/[4(*τ*_*peak*_ + *τ*_Δ*I*_)]. With the peak time *τ*_*peak*_ = 0.53 ms read off from the cross-correlation curve, this procedure yields 5.7 · 10^−8^ cm^2^/s, in reasonable agreement with the value originally assumed in the numerical simulation of 4.5 · 10^−8^ cm^2^/s. It should be noted that in a tight focus the hot spots are somewhat displaced from the holes, leading to a reduced separation (110 nm rather than 120 nm), and concomitantly a derived *D* = 4.8 · 10^−8^ even closer to the assumed value. Hence, the alternating nano-aperture array geometry indeed allows measurement of diffusion constants through sample geometry, circumventing the need for calibration runs on known solutions. In principle the need to precisely calibrate the shape of the *MDF* is obviated by the fact that aperture spacing is a robust calibration-free ruler. Our simulations show that this robustness improves when using a less tight illumination focus. In this case the fact that more apertures are illuminated removes the dependence on where the center of the focus actually is chosen (which leads to the difference between ACF_*x*_ and ACF_*y*_ in Fig. 3e), and the hot spot spacing more closely approaches the sample periodicity.

## Experiment

**Figure 4:**
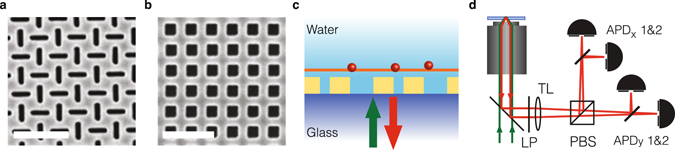
Proof of principle experiment for nano-aperture enhanced fluorescence correlation spectroscopy. (**a,b**) SEM images of a polarization sensitive nano-aperture array of rectangular 165 by 50 nm slits at 180 nm pitch (**a**) and a polarization insensitive reference array of 100 × 100 nm^2^ square apertures at 200 nm pitch (**b**). Scalebars indicate 500 nm. (**c**) In the experiment the gold film is covered by a 30 nm SiO_2_ coating (spincoated HSQ) which supports a lipid bilayer. Emitters are diffusing in the plane of the lipid bilayer. The sample is immersed in water, and we pump and collect emission from the glass side of the sample. (**d**) We study a lipid bilayer sample in a confocal fluorescence microscope. Light collected from the emitters is passed through a longpass (LP) filter, and split into polarization channels by a polarizing beam splitter (PBS). We use two APDs per polarization channel to avoid artefacts due to APD deadtime and afterpulsing.

We have performed a proof of principle experiment, using focused ion beam milling to make nano-aperture arrays in thermally evaporated gold films (100 nm thickness) on glass which was coated with a planarizing layer of 30 nm SiO_2_ spin-on glass (HSQ) after milling. We have fabricated arrays of rectangular arrays of 165 nm length, 50 nm width, and arranged on a grid of 180 nm. As a reference system, we fabricated arrays of pitch 200 nm and square 100 nm holes. Since these holes are square, no polarization sensitivity is expected. Figure 4(a,b) shows electron microscopy images of the fabricated arrays. On the metal film we prepared a supported lipid bilayer composed of DOPC (*L*-a-phosphatidylcholine, Avanti Polar Lipids) doped with nominally 0.5 · 10^−6^ mol % Rho-PE (*1*,2-dipalmitoyl-sn-glycero-3-phosphoethanolamine-N-(lissamine rhodamine B sulfonyl) ammonium salt, Avanti Polar Lipids) to perform FCS on their 2D diffusion.

We obtained FCS traces using a homebuilt fluorescence microscope (see Fig. 4(c,d)). The sample was pumped with 300 *μ*W using the 568 nm line of a cw Ar:Kr laser. Excitation and collection were performed through the glass side of the sample, using a Nikon CFI S Plan Fluor 60x ELWD objective (NA 0.7). For detection we pass the light through two Chroma HG580LP filters to reject laser light, and onto Si avalanche photodiodes (APDs) in Geiger mode (Micro Photon Devices). The APDs are connected to a 16-channel Becker and Hickl DPC230 time-correlator card in time tagging mode. Photon time traces of 120 seconds were correlated using in-house developed software using the multi-tau algorithm^34^. To improve statistics we average three correlation traces. We use a polarizing beam splitter to separate out the two polarization channels. To avoid correlation artefacts that may appear in autocorrelation traces due to APD dead times, we divide the signal over two APDs in each polarization branch.

**Figure 5:**
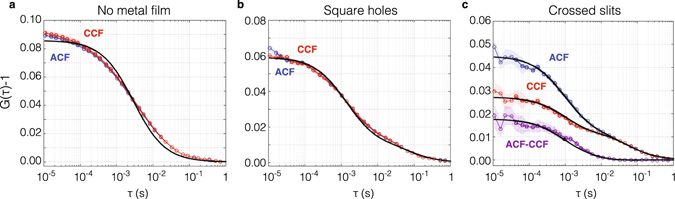
Experimental intensity correlations for nano-aperture 2fFCS. Intensity correlations for (**a)**. A lipid bilayer directly on glass, (**b**). A polarization insensitive square hole array in gold, and (**c**). The crossed slits. Blue curves with circular symbols correspond to autocorrelations, i.e., correlating detectors in the same polarization channel, while red curves with square symbols correspond to cross-correlation of polarization channels. For the reference system (**a**) and the polarization insensitive antennas (**b**), the auto- and crosscorrelates are identical, but a strong difference appears for the crossed nanoslit sample (**c**). Shaded areas around the curve indicate the standard error in the mean, given that curves are averages over 3 runs of 120 seconds each, where we furthermore average over all auto- resp. crosscorrelating detector combinations. Dotted (dash-dotted) black lines indicate the fitted ACF (CCF) according to a simple Gaussian model (see text). Note that for panels b,c the difference is the polarization cross-talk required to fit the data. In (**b**) all apertures contribute equally independent of polarization leading to an identical auto- and crosscorrelation. In (**c**) the *x*(*y*)-oriented holes contribute 4 times more strongly to *MDF*_*y*(*x*)_ than the *y*(*x*)-oriented holes. In panel c the purple curve shows the difference in correlations, corresponding to the temporal correlation of instantaneous polarization differences. The black curve superimposed on the purple curve is the difference of the fits to the CCF and ACF, which has a rolloff time of 0.93 ms. Diffusion at the aperture substrate is slowed down compared to the reference case (*D* = 0.33 versus 4.5 *μ*m^2^/s).

Figure 5 shows the obtained FCS traces. Panel (a) pertains to a reference measurement without gold film, while panel (b) and (c) pertain to the arrays of square resp. rectangular holes. For the reference sample of square arrays, both polarization channels are expected to correspond to identical molecular detection functions. Indeed, Fig. 5(b) shows that the cross-correlation between polarization channels gives a time-trace identical to the autocorrelation of each polarization channel separately. Instead, for the array of alternating rectangular nano-apertures, the standard FCS trace (single polarization channel) and the cross-correlation of cross-polarized channels shows a large difference (Fig. 5(c)). This measurement demonstrates the feasibility of nano-antenna 2fFCS. We emphasize that all detectors are confocal with the (same) diffraction limited sample excitation spot, which encompasses about five optically unresolvable nanoapertures in total. The cross-correlation contrast hence entirely comes from the encoding of spatial information, i.e., fluorophore location, in polarization channels.

The fact that the experimental data shows a high value of the cross-correlation at *τ* = 0 indicates significant spatial overlap of the two *MDF* s, which we attribute to non-perfect polarization contrast in the nanoapertures. Indeed, similar curves are observed in simulations similar to those in Fig. 3, for geometries and operating wavelengths that do not result in strong polarization separation. As a result of the resulting cross-talk between polarization channels, there is no clear peak in the cross correlation time trace as there would be in the ideal case presented in Fig. 3(e). However, one can still fit the time traces to obtain diffusion constants. For verification we first fit a single gaussian FCS model to reference data without a gold film (Fig. 5(a)). If we assume a focus width of 405 nm (intensity FWHM), tantamount to an MDF width *σ* = 243 nm^23^, we find a diffusion coefficient of 5 *μ*m^2^/s, in reasonable agreement with a previously reported value of 4.5 *μm* ^2^/s^22, 35^. Thereby, this fit verifies the operation of our set up, and our focus size estimate in absence of the plasmonic structures.

We continue by globally fitting a numerical model to the auto and cross-correlation data of the arrays. This model is based on ref. 23 which treats FCS in focal distributions that are a superposition of many Gaussian contributions. We modified this model to deal with 2D systems in which we assume an MDF is given by the sum of a broad background focus, and a periodic array of hot spots spaced by the array pitch in our sample. The hot spot amplitudes are a multiple of the local background focus intensity, where the enhancement factor and hot spot size are treated as two fit parameters. We further extended our model to compute auto- and cross-correlations of different sets of gaussian volumes. The polarization-selective behavior is implemented by assigning an amplitude difference to sets of orthogonally oriented apertures that reverses between *MDF*_*x*_ and *MDF*_*y*_. This model allows us to efficiently calculate the auto- and crosscorrelations for complex MDFs accounting for up to 100 holes, using just seven parameters: five to define the geometry (background focus size, hot spot spacing, hot spot size, hot spot enhancement factor, polarization contrast) and two to quantify the fluorophore physics (concentration and diffusion constant). We have verified that this model can successfully reproduce simulated correlation curves such as the ones shown in Fig. 3e.

To further constrain the fit, we fix the background focus size to 405 nm from the calibration measurement, and the hot spot spacing to the 180 nm sample pitch. We simultaneously fit the square-hole and rectangular-hole ACF and CCF data traces, imposing identical concentration and diffusion constant. Even with these tight constraints, the model satisfactorily reproduces our data, precisely tracing both the cross- and autocorrelations. We note that this fit is obtained with a diffusion coefficient of 0.33 *μm* ^2^/s, significantly below the value obtained in the reference system. We attribute this discrepancy to a difference in electrostatic properties between the glass reference, and the aperture array, due to surface properties of the gold film and planarization layer, and their modification by focused ion beam milling. Importantly, this discrepancy has no bearing on the validation of the optical mechanisms of nano-antenna enhanced 2fFCS per se, as is evidenced by the values for the geometrical fit parameters that we retrieve. According to the fit, hot spot sizes are approximately 55 nm FWHM for the rectangular resp. square hole samples, with MDF-enhancement factors in the hot spots a factor 12 resp. 6 compared to the background. The polarization contrast in the rectangular sample is approximately 4:1 according to the fit. Figure 5c also shows the roll off of the polarization fluctuations, i.e., the measured difference between ACF and CCF data sets, overplotted with the difference of the fit curves. The roll off time of 0.93 ms directly translates to a hot spot size *σ* = $\sqrt{4D\tau }$ = 35 nm (translates to FWHM 55 nm), using the diffusion constant fitted to the rectangular and square datasets. This size is in good agreement with the simulated hot spot sizes.

We conclude that our experiment supports the proposition of nanoantenna 2fFCS. In this proof-of-concept experiment, the cross-correlation peaks at zero time delay as opposed to the distinct peak at non-zero time delay in Fig. 3(e). This indicates that in our experimental realization there is still substantial overlap between the cross-polarized *MDF* s. Indeed, our fit indicates cross-talk through the background focus, and through the fitted cross-polarization contrast of 4:1, indicating that’dark’ cross-polarized slits still contribute to the *MDF* with 25% of the strength of co-polarized ‘bright’ slits. We anticipate that these shortcoming can be resolved by improved fabrication procedures and a sample design that is more tailored to align plasmon resonance and emission properties. A more red-shifted dye than tetramethylrhodamine (fluorescence at 576 nm), or a different plasmonic metal to blueshift the plasmon resonance would be helpful to align the emission with the slit resonance. Moreover, low-quantum efficiency dyes are well-known to be much more effective at singling out hot-spot properties^36^, likely leading to improved polarization contrast by removing the background focus contribution.

## Conclusion and outlook

We have shown a nano-antenna design which encodes fluorescence emission originating from different spatial regions into two orthogonal far-field polarization states with high contrast. It combines the advantages of calibration free 2fFCS measurement and the benefits of nano-antenna enhanced fluorescence spectroscopy, like smaller detection volumes, and pump and emission enhancements. The use of polarization sensitive nano-antennas to provide spatial selection reduces the complexity of 2fFCS experiments compared to existing far-field implementations. In the proposed nano-optical implementation, only the addition of a polarization splitter and detection path is necessary. Otherwise, the set up remains entirely identical to a single-focus confocal set up, as opposed to having to create displaced foci.

The discussed geometries are a proof of principle and are not yet completely optimized. The general design rules identified in this work are that (i) polarization cross-talk between MDF channels must be minimized, while simultaneously (ii) no background focus contribution must be present. In this work we followed the rationale that polarization cross-talk is minimized by minimizing the dipole-dipole coupling between plasmon resonances by spatial arrangement. Our full-wave simulations show that this philosophy can lead to robust designs even at small spacings between slits. Large gains in performance could be made by combining these design philosophies with established methods to increase the light collection efficiency. The periodic array can for instance further be tailored to ensure that light that is emitted into the SPP guided mode is beamed into the far-field, thereby increasing the count rate per fluorophore^7^. Also, local density of states enhancements can be used to boost count rates per molecule, and to make molecular detection functions more spatially selective^10, 11, 36^. Furthermore, other types of far-field channels could be used to address different near-field volumes. For example, from ref. 37 we extrapolate that dividing the radiation pattern in different detection channels allows to select distinct near-field volumes around complex nano-structures with sub-diffractive spacing.

### Data availability

The datasets generated during and/or analysed during the current study are available from the corresponding author on reasonable request.
